# A Critical Reexamination of Recovered SARS-CoV-2 Sequencing Data

**DOI:** 10.1093/molbev/msaf109

**Published:** 2025-06-09

**Authors:** Florence Débarre, Zach Hensel

**Affiliations:** Institute of Ecology and Environmental Sciences, CNRS UMR 7618, Sorbonne Université, UPEC, IRD, INRAE, Paris, France; Instituto de Tecnologia Química e Biológica, Universidade Nova de Lisboa, Av. da República, 2780-157 Oeiras, Portugal

**Keywords:** SARS-CoV-2, COVID-19, sequence read archive, phylogenetics, data recovery, metadata, epidemiology

## Abstract

In 2021, Jesse Bloom published a study addressing why the earliest SARS-CoV-2 sequences in Wuhan from late December 2019 were not those most similar to viruses sampled in bats. The study concluded that recovered partial sequences from Wuhan and annotation of Wuhan links for other sequences increased support for one genotype as the progenitor of the SARS-CoV-2 pandemic. However, we show that the collection date for the recovered sequences was January 30, 2020, later than that of hundreds of other SARS-CoV-2 sequences. Mutations in these sequences also exhibit diversity consistent with SARS-CoV-2 sequences collected in late January 2020. Furthermore, we found that Wuhan exposure history was common for early samples, so Bloom's annotation for a single familial cluster does not support that an early genotype was undersampled in Wuhan. Both the recovered partial sequences and additional annotation align with contemporaneous data rather than increase support for a progenitor. Our findings clarify the significance of the recovered sequences and are supported by additional data and analysis published since mid-2021.

## Introduction

In 2021, Jesse Bloom published an analysis of early SARS-CoV-2 sequences (Bloom 2021) that included data generated from samples collected at Renmin Hospital of Wuhan University (Wang et al. 2020b). Bloom concluded that these data increased the plausibility of one genotype as the progenitor of SARS-CoV-2. The data were generated during the development of a diagnostic method based on sequencing partial SARS-CoV-2 genomes using nanopore technology (Wang et al. 2020b). Primarily aimed at detecting infections by SARS-CoV-2, the technique could also be used for genotyping. Wang et al. first shared their results in a preprint in March 2020 (Wang et al. 2020a). They submitted sequencing data a few days later to the sequence read archive (SRA; see supplementary table S1, Supplementary Material online for a timeline). The work was submitted to the journal *Small* in April, revised in May, and published in June 2020 (Wang et al. 2020b). The article included in its Table 1 a list of mutations identified in samples, and grouped samples in what was later described as lineage A and lineage B (Rambaut et al. 2020; Tang et al. 2020). Following *Small*’s format at the time, the article did not include a data availability statement (Wang et al. 2021; Zimmer 2021). The data depositor requested that sequencing data be withdrawn from the SRA. Withdrawal should have just excluded the data from search results, but instead the data were deleted (Berman et al. 2022; Brunak et al. 2002). Some relevant details about this particular issue can be found in supplementary table S1, Supplementary Material online, and in a preprint version of our work (Débarre and Hensel 2024). However, the deletion did not impact Bloom’s data or analyses, on which we focus here.

In 2021, Jesse Bloom discovered Wang et al.’s sequencing data via another study on early pandemic sequencing data (Bloom 2021; Farkas et al. 2020). Bloom recovered the sequencing data from NCBI Google Cloud Storage and from a mirror of SRA data (Bloom 2021; Lifebit 2020). In his study, Jesse Bloom explored possible roots of the early SARS-CoV-2 phylogeny on a set of sequences collected before February 2020. He addressed a well-known conundrum (Rambaut et al. 2020), illustrated in supplementary fig. S1A, Supplementary Material online, that he summarized as: “the earliest reported sequences from Wuhan are *not* the sequences most similar to SARS-CoV-2’s bat coronavirus relatives” (Bloom 2021). Using the principle of outgroup rooting (rather than molecular clock rooting that accounts for collection dates), Bloom manually rooted phylogenetic trees of early SARS-CoV-2 sequences at three nodes most similar to a bat-virus outgroup (see supplementary table S2, Supplementary Material online). Next, Bloom qualitatively inferred the relative degree of support for each root by considering the locations and dates at which samples were collected, identifying “two plausible progenitor sequences.” The third proposed progenitor (A+C3171T) was considered less plausible because “it has almost no weight from Wuhan and the first sequence identical to its progenitor was not collected until January 24[, 2020].” The A+C18060T progenitor was kept because one sequence was sampled in Wuhan, although it was collected on January 26, 2020. For A+C29095T, Bloom noted the presence of an A+C29095T sequence among the Wang et al. data (positions 3,171 and 18,060 were not covered in these sequences, so Wang et al.’s data cannot inform on the plausibility of the corresponding proposed roots; see supplementary table S2, Supplementary Material online), and he annotated four sequences collected on January 10 to 15 from patients in Guangdong province with Wuhan travel history (Chan et al. 2020; Kang et al. 2020). Bloom distinguished the recovered sequences and the Guangdong patient sequences from the broader pre-February dataset by annotating them in figures as “deleted early Wuhan” and “Guangdong patient infected in Wuhan before January 5” (respectively; see supplementary fig. S1B, Supplementary Material online). The reported collection dates of recovered sequences were described as inconsistent with Wang et al.’s (2020b) text (“on or after January 30, 2020, *rather than* ‘early in the epidemic’ as originally described in Wang et al. (2020b)”; Bloom 2021, emphasis added). Bloom further wrote that it was “impossible to […] determine exactly when *[the recovered sequences]* were collected.” Uncertainty in collection dates and the possibility that they could be among the “earliest” samples was also communicated in the popular and scientific news media (Chang and Langreth 2021; Cohen 2021; Pease 2021).

While compiling sequencing data and metadata for another project, we found that January 30, 2020 sample collection dates for Wang et al. (2020b)’s sequences were available on the SRA. According to the SRA team (email to FD, December 2023), these collection dates are identical to those provided by Wang et al. in March 2020. Here, we provide multiple lines of evidence showing that the January 30, 2020 collection date was correct. We show that the datasets from which Bloom obtained Wang et al. (2020b)’s data included collection date metadata (Farkas et al. 2020; Lifebit 2020). We additionally show how Guangdong patient sequences with A+C29095T are linked to the same Wuhan hospital, and that this genotype is not unique in its association with Wuhan. Lastly, we discuss how robust pandemic origins scenarios must rigorously account for available data, including sample collection dates, available metadata, and refined estimates of mutation rates.

## Results

### The January 30, 2020 Collection Date was Present in the Dataset from Wang *et al.* (2020)

**Fig. 1.**
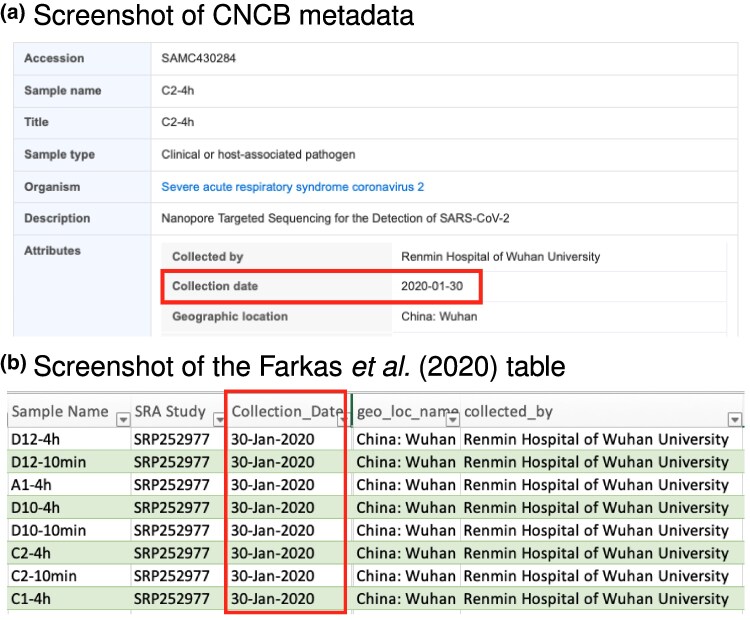
The 30-January collection dates were available to Bloom and unchanged since first published in March 2020. a) Screenshot of metadata on CNCB (https://ngdc.cncb.ac.cn/biosample/browse/SAMC430284). b) Screenshot of metadata in Farkas et al. (2020) table (https://dfzljdn9uc3pi.cloudfront.net/2020/9255/1/Supplementary_Table_1.xlsx; https://peerj.com/articles/9255/#supp-2; the file is also available in Jesse Bloom’s Github repository at https://github.com/jbloom/SARS-CoV-2_PRJNA612766/blob/main/manual_analyses/PRJNA612766/Supplementary_Table_1.xlsx). The file was available since 2020 and was used by Bloom to discover the Wang et al. sequencing data. Bloom replaced the date in his https://github.com/jbloom/SARS-CoV-2_PRJNA612766/blob/main/manual_analyses/PRJNA612766/extract_accessions.ipynb script.

In his article, Bloom claimed that the recovered sequences “lack[ed] full metadata,” and that as a result it was “impossible to […] determine exactly when they were collected.” However, while Bloom cited a press conference and two blog posts indirectly reporting January 30, 2020 collection dates (Wang 2021a, 2021b), collection dates were published earlier in July 2021 in a dataset cited by Bloom (PRJCA005725; Fig. 1a). The same date is present in the Farkas et al. (2020) table (Fig. 1b), from which Bloom identified Wang et al. (2020b) sequencing data. We further found the same collection dates in another dataset used in Bloom’s study, a mirror of early pandemic sequencing data from Lifebit Life Sciences (Lifebit 2020). Bloom replaced collection dates of January 30, 2020 by “early in epidemic” while processing metadata during data analysis in a script listed in his Materials and Methods section, and did not report the collection dates. Ultimately, this resulted in labeling the recovered sequences as “deleted early Wuhan” (in his Fig. 5) rather than showing their collection date.

Following the logic of Bloom’s paper, a January 30, 2020 collection date is too late to shift the likelihoods of proposed SARS-CoV-2 progenitor genotypes. For instance, the third proposed root (A+T3171C) was considered less plausible by him in part because “the first sequence identical to its progenitor was not collected until January 24.” In addition, full genome sequences from samples collected before February 2020 were not rare: there were 507 such sequences in data considered by Bloom (2021). Wang et al. (2020b)’s sequences are therefore not exceptional.

Collection dates on or after January 30, 2020 have consistently been in Wang et al. ’s sample metadata since March 2020. There is no evidence that this collection date could be inaccurate. This type of information is commonly extracted from metadata. Finally, collection dates being unreported or imprecisely described in corresponding scientific papers (or the lack of a corresponding paper) was not an exclusion criteria for other sequences in Bloom’s study (e.g. his inclusion of late January Wuhan sequences from Yan et al. 2021). Nonetheless, occasional errors in chronological metadata have complicated accurately inferring phylogenetic trees during the pandemic (e.g. Sanderson 2024). We therefore investigated whether the composition of recovered sequences is consistent with January 30 collection dates, with analyses limited to Bloom (2021)’s dataset (i.e. sequences available in mid-2021).

### Recovered Sequences are Consistent with Contemporaneous Data, Supporting Late January Sample Collection

We compared the data from Wang et al. to contemporaneous data in two different ways. First, we turn to Wuhan sequencing data generated via a similar nanopore-based technology as Wang et al. (2020b), reported in the context of an article by Yan et al. (2021). The samples were collected from “various Wuhan health care facilities” on January 25, 2020 and 26; consensus sequences were deposited on GISAID; they are included in Bloom’s study. Two sequences from the Yan et al. (2021) dataset are present in proposed progenitor nodes in Bloom (2021): C13 in the A+C18060T root, and C31 in the A+C29095T root.

**Fig. 2.**
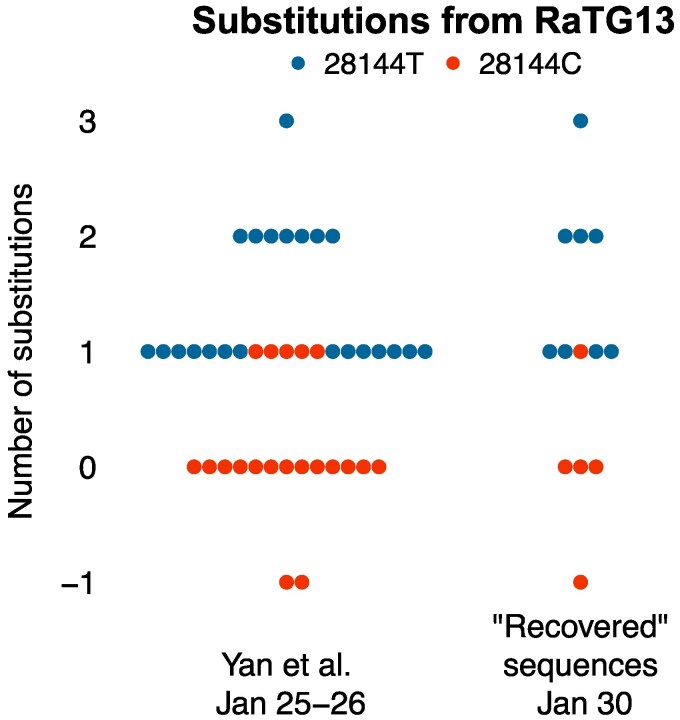
Number of substitutions from bat SARS-like coronavirus RaTG13 in the region between nucleotides 21,570 to 29,550 that is considered in Fig. 4 in Bloom (2021) [relative to lineage A, which is equivalent to lineage A+C18060T {“proCoV2”} in this region, as position 18,060 is not covered]. Sequences from Yan et al. (2021) are compared to those from Wang et al. (2020b) (recovered sequences). Substitutions are counted such that 0 corresponds to the same distance as between RaTG13 and lineage A; negative values (−1) correspond to additional substitutions towards RaTG13 (C29095T for a recovered sequence and for one of the Yan et al. (2021) sequences, and C22747T for the other Yan et al. sequence). Substitution T28144C is characteristic of lineage A and is highlighted in red. (NB: We use RaTG13 only for the sake of comparison with Bloom’s analysis.)

The distribution of substitutions in sequences from Yan et al. (2021) is similar to that of Wang et al. (2020b) (Fig. 2). In particular, the proportions of the lineage-A defining mutation T28144C are indistinguishable in the two datasets [17/42 in the Yan et al. (2021) data and 5/13 in the recovered sequences; Fisher’s Exact Test, P=1]; so are also the proportions of the C29095T mutation highlighted by Bloom [1/42 in the Yan et al. (2021) data and 1/13 in the recovered sequences; Fisher’s Exact Test, P=0.42]. The two distributions remain similar when the outgroup comparator is changed (supplementary fig. S2, Supplementary Material online).

Substitutions towards the chosen outgroup are not necessarily signs of their ancestral nature. The −1 positions of three sequences in Fig. 2 are due to C29095T (one recovered sequence and one sequence from Yan et al. 2021) and to C22747T (the other Yan et al. 2021 sequence). Both substitutions have subsequently reappeared in other SARS-CoV-2 lineages (see supplementary fig. S3, Supplementary Material online). Outside of the region covered in sequences from Wang et al., the Yan et al. sequence with C22747T also contains T4402C and G5062T, identifying C22747T as a reversion subsequent to mutations that characterize a common early epidemic genotype in lineage A.

The comparison can be extended to the whole dataset used by Bloom (2021) (supplementary fig. S4, Supplementary Material online). Focusing on sequences collected ± 7 d around January 30, 2020, the proportions of key mutations such as T28144C and C29095T are, again, indistinguishable between Bloom’s dataset and the recovered sequences (T28144C: 226/650 in Bloom (2021)’s dataset; Fisher’s Exact Test, P=0.78; C29095T: 30/650; Fisher’s Exact Test, P=0.47). Given the proportion of C29095T in the available sequences (4.6% in Bloom’s dataset), there was a high chance of finding this specific mutation in at least one of the 13 additional sequences from Wang et al.. The recovered sequences dataset is, therefore, unremarkable; it is consistent with expectations for samples collected in Wuhan around January 30, 2020. Thus, identifying one example of A+C29095T in the recovered sequence dataset does not support the conclusion that A+C29095T was underrepresented in the earliest sequences.

### The Guangdong Sequences with a Wuhan Exposure Were not Independent

In addition to identifying A+C29095T in one of Wang et al. (2020b)’s sequences, Bloom (2021) argued that A+C29095T was also supported as a progenitor by “many of the sequences […] from early patients who were infected in Wuhan but then sequenced in and attributed to Guangdong.” This Wuhan link in a well-described cluster (Chan et al. 2020; Kang et al. 2020) had previously been identified in an early analysis of possible progenitor genotypes (Yu et al. 2020). Initially described by Bloom (2021) as “two different clusters of patients who traveled to Wuhan in late December of 2019,” a correction now notes that there was one cluster rather than two (Bloom 2023), after we and others pointed it out. The change is significant, because it decreases the corresponding estimated prevalence of A+C29095T in Wuhan. We identified additional sequences from this cluster, sometimes multiple samples from the same patients, that were included in Bloom’s dataset without this annotation (see supplementary tables S3 and S4, Supplementary Material online for details). Further, we note that patients in the cluster did not just travel to Wuhan in late December 2019, but had visited a relative hospitalized in Wuhan for febrile pneumonia (Chan et al. 2020). In other words, they had been to one of the few places other than the Huanan market where one was most likely to encounter people infected by SARS-CoV-2 in Wuhan at that early date.

Analyzing early exports from Wuhan might characterize the Wuhan outbreak with less risk of ascertainment bias. We find that epidemiological links to Wuhan are very common in case reports from January 2020, and are not limited to A+C29095T sequences. Many other sequences in Bloom’s trees were from direct exports from Wuhan, but were not labeled as such (see our annotations in supplementary fig. S1C, Supplementary Material online). For example, all eight sequences in Bloom’s proposed A+T3171C root have a documented epidemiological link to Wuhan (Jiang et al. 2020), as does the first Covid-19 case detected in the United States with A+C18060T (Holshue et al. 2020). This is also true of early international exports of lineage A (Eden et al. 2020) and lineage B (Okada et al. 2020). Importantly, the earliest known export is a lineage B case with symptom onset predating any case in the Guangdong cluster by almost 2 weeks, and it is linked to the Huanan market (Liu 2020).

### New Data Published Since Mid-2021 Fail to Support an A+C29095T Progenitor

First, we checked whether our conclusions still held using a second dataset compiled using stringent quality control (Pekar et al. 2022), to which we added recently published sequences (Lv et al. 2024), totaling 448 sequences collected before February 2020. We find that the number of substitutions in the Wang et al. (2020b) dataset remains consistent with those observed in other sequences with similar collection dates (supplementary figs. S5 and S6, Supplementary Material online).

We also investigated whether sequences reported by Lv et al. (2024) would support Bloom’s conclusions, which were based on identifying early Wuhan sequences with the proposed progenitor genotypes. None of the eight high-coverage sequences collected by Lv et al. (2024) before February 2020 would fall into a progenitor node in Bloom’s phylogenetic trees: the one sequence with C29095T, collected on January 29, 2020, also has G25947T and would therefore be placed in another, derived, node with sequences from samples collected in Wuhan and Shanghai. We found that another Lv et al. (2024) sequence with C29095T, sampled on February 3, 2020, had an identical genotype and comparable metadata to an already existing sequence from Shanghai. Further inspection showed that early sequences in Lv et al. (2024) were independent samples obtained from the same cohort of patients (laboratory-confirmed COVID-19 patients hospitalized at Shanghai Public Health Clinical Center between January 20, 2020 and February 25, 2020) as another study (Zhang et al. 2020). Lv et al. (2024)’s sequences therefore do not bring support to Bloom (2021)’s conclusions.

Lv et al. (2024) also report direct Wuhan links for 8 of 13 Shanghai patients with epidemiological history and collection dates before February 2020. This further emphasizes the need to thoroughly annotate epidemiological links rather than draw conclusions from partial annotation.

The history of the Guangdong cluster indicated that the C29095T substitution was present in Wuhan in late December 2019; it is therefore unsurprising that C29095T was detected in late January 2020 in Wuhan by Yan et al. (2021) and by Wang et al. (2020b). Methods in Bloom (2021) did not account for the fact that C→T mutations are by far the most frequent type of mutation during the pandemic (Azgari et al. 2021; De Maio et al. 2021). Subsequent work on this topic (Bloom et al. 2023; Ruis et al. 2023) has even specifically identified C29095T as occurring much more frequently than a typical C→T mutation (Bloom and Neher 2023, supplementary data nt_fitness.csv). Of all mutations considered by Bloom and Neher (2023)(three possible SNPs at each of 29295 positions), C29095T is the ninth most frequent one, making it more likely to be derived than ancestral compared to the other potential progenitors considered by Bloom (2021). In fact, C29095T recurs in Bloom’s phylogenetic trees, where this position mutates three times.

Finally, Bloom (2021) noted that all sequences linked to the Huanan market were of lineage B, three mutations away from his proposed progenitors. At the time, this observation had led to the suggestion that the market had been a place of secondary amplification, but not the source of the outbreak. Later analyses and data challenged this conclusion. The two lineage-A cases with onset in December 2019 were shown to be geographically associated with the market (Worobey 2021; Worobey et al. 2022), leading to the prediction that lineage A was in the market. Then, lineage A was detected in an environmental sample from the market (Liu et al. 2024): in a stall with a suspected case with mid-December 2019 onset, and trace evidence in one sample from a different stall (Crits-Christoph et al. 2024). Lastly, the predominance of lineage B among Wuhan samples was observed beyond potential ascertainment bias linked to the Huanan market. A study genotyping random samples from Wuhan patients admitted to five hospitals found a consistent predominance of lineage B (Hu et al. 2021), mirroring what was observed for early pandemic sequences collected in Wuhan and also globally.

## Discussion

The facts that we present do not support Bloom (2021)’s conclusion that Wang et al. (2020b)’s sequences demonstrate that A+C29095T was underrepresented in the earliest sequences, nor his conclusion that they increase the plausibility of A+C29095T as the progenitor genotype of SARS-CoV-2. Wang et al. (2020b)’s samples were collected in late January 2020, and mutations identified in these samples, including C29095T, are unsurprising to find again in Wuhan. Further, links to Wuhan were common, and annotating them just for sequences in one cluster does not distinguish the A+C29095T sequences as more likely to be ancestral than others.

There was no contradiction in Wang et al. (2020b)’s 2020 and 2021 statements on collection dates: the date they published in 2021 (January 30, 2020) was the same as the date they had submitted to the SRA in March 2020, and the term “early in epidemic” is not inconsistent with the date. The meaning of “early in the epidemic” is context-dependent: “early” at Renmin Hospital is later than at hospitals closer to the epicenter of the outbreak, but likely earlier than at hospitals outside of Wuhan. Wang et al. (2020b) used samples collected while fever clinics at Renmin Hospital were suddenly overwhelmed with demand for molecular testing of suspected Covid-19 1 week after the beginning of Wuhan’s lockdown (Liu et al. 2020) and only a few days after Renmin hospital was still sending staff to supplement Jinyintan hospital where the earliest patients were sent for treatment (Hubei Daily 2020; Yang 2024). “Early,” however, does not necessarily mean “the earliest.” Late January is not so early that the Wang et al. (2020b) sequences can support the conclusion that A+C29095T was underpreresented in the earliest samples collected from Covid-19 patients. It is also critical to understand the context in which scientists in Wuhan were working in early 2020. While Bloom (2021) criticized Wang et al. (2020b) for not fully sequencing their samples, the fact that full genome sequencing was not prioritized in late January 2020 in Wuhan realistically reflected prioritizing capabilities for patient diagnosis, isolation, and treatment.

Although partial SARS-CoV-2 sequences are of limited value for phylogenetic studies, the data shared by Wang et al. (2020b) contained information. The mutations reported in Wang et al. (2020b)’s Table 1 include additional mutations from early SARS-CoV-2 lineages, notably one mutation (A24325G, sample B9) found in a sample from a Huanan market vendor (World Health Organization 2021). In addition, other Wang et al. samples are in sublineages derived from lineage A and lineage B, indicating that these are not samples from patients with the earliest infections of the pandemic. To our knowledge, no subsequent phylogenetic analysis since Bloom (2021)’s paper has used Wang et al. (2020b)’s data. Had assembled versions of Wang et al. (2020b)’s sequences been shared on GISAID, they would have been excluded in Bloom (2021)’s analysis, because of their partial coverage.

The question of the precise identity of SARS-CoV-2’s root remains unresolved. Using a method that accounts for both sample collection dates and the sequences of related bat coronaviruses, lineage A without additional mutations was identified as the most likely genotype of the common ancestor of SARS-CoV-2 sequences from humans (Crits-Christoph et al. 2024; Pekar et al. 2022). This analysis strongly rejects A+C18060T and A+C29095T as ancestral haplotypes (Crits-Christoph et al. 2024, supplementary Table S1, Supplementary Material online). To address the rooting conundrum, Pekar et al. (2022) considered another SARS-CoV-2 origin scenario, involving multiple SARS-CoV-2 spillovers from animals to humans, with lineage A spillover likely occurring after lineage B. Such a scenario of multiple transmissions close in time and space, from a group of animals to humans, also occurred later in the pandemic (AHAW et al. 2023), notably with pet hamsters in Hong Kong, for which a genomic investigation identified multiple zoonotic spillovers (Yen et al. 2022). Low diversity in coronavirus genomes identified in samples from bats at the same time and place is also common; for example, RshSTT182 and RshSTT200 genomes differ by only three nucleotides (Delaune et al. 2021).

Bloom (2021) addressed the conundrum that “more ancestral” genotypes are not among the SARS-CoV-2 sequences with the earliest collection dates in Wuhan, by considering that an A+C29095T or A+C18060T progenitor may be underrepresented because of uneven sampling and/or reporting. We have shown that additional evidence considered by Bloom—the identification of one A+C29095T sequence collected in Wuhan on January 30, 2020, and the annotation of a Wuhan link for four sequences in one cluster—does not actually increase support for an A+C29095T progenitor. Our analysis was robust when considering recently published sequences (Lv et al. 2024). It is critical that conclusions be continuously tested by considering all available data, including sample collection dates, and justifying (meta)data exclusion.

## Materials and Methods

We followed the same methods as Bloom (2021) to compare sequences to outgroups.

### Source Data

We used data shared by Bloom on Github at https://github.com/jbloom/SARS-CoV-2_PRJNA612766.

For the expanded dataset, we used outputs of a dataset curated by Pekar et al. (2022), complemented by data recently shared by Lv et al. (2024) (selecting the earliest informative sequence for each patient as described in Crits-Christoph et al. (2024); Patients IDs are provided in Lv et al. (2024)’s appendices. Selected accessions are listed in the associated Zenodo repository.) We gratefully acknowledge the authors from the originating laboratories and the submitting laboratories, who generated and shared through GISAID the viral genomic sequences and metadata on which this research is based. GISAID accessions used are the same as Pekar et al. (2022) data S1. The Yan et al. (2021) data correspond to EPI_ISL_493149 to EPI_ISL_493190 and are included in the data analyzed by Bloom (2021).

Collection dates are available in SRA metadata in the Collection_Date field.

### Rank of C29095T

The rank of rate of C29095T substitution was extracted from the results of a previously reported analysis (Bloom and Neher 2023) using an updated analysis from 06-Nov-2024 (commit 067fce1) https://github.com/jbloomlab/SARS2-mut-fitness/blob/main/results/nt_fitness/ntmut_fitness_all.csv. In this analysis, the frequencies of occurrences of individual SNPs are extracted from the UShER tree of public SARS-CoV-2 sequences (Turakhia et al. 2021), excluding a small fraction of masked sites and likely sequencing artifacts. We ranked SNPs by the number of occurrences. The corresponding code is available in the Zenodo repository.

## Supplementary Material

msaf109_Supplementary_Data

## Data Availability

Data and code are available on Zenodo (https://zenodo.org/doi/10.5281/zenodo.10665464). We used the same sequence datasets as Bloom (2021) (accessions listed in https://github.com/jbloom/SARS-CoV-2_PRJNA612766/blob/main/results/early_sequences/deltadist.csv). Additional analysis with more recent data used the same accessions as Crits-Christoph et al. (2024) (listed in data/accessions_P22-Lv24.csv in the Zenodo repository.) The Wang et al. (2020b) sequences are available in project PRJCA005725 on CNCB.
