# Supplementary material for: A Critical Reexamination of Recovered SARS-CoV-2 Sequencing Data
Source: Mol Biol Evol. 2025 Jun 9;42(6):msaf109. doi: 10.1093/molbev/msaf109 (PMC12147218; doi:10.1093/molbev/msaf109)
Supplement: msaf109_Supplementary_Data [file msaf109_supplementary_data.zip › Debarre-Hensel_MBE_SuppInfo.pdf]

Supplementary materials for

A critical reexamination of recovered SARS-CoV-2 sequencing data

F. Débarre[1] & Z. Hensel[2]

[1] Institute of Ecology and Environmental Sciences, CNRS UMR 7618, Sorbonne Université, UPEC, IRD, INRAE, Paris, France
https://orcid.org/0000-0003-2497-833X
[2] ITQB NOVA, Universidade NOVA de Lisboa, Lisbon, Av. da República, 2780-157, Oeiras, Portugal
Corresponding author: florence.debarre@normalesup.org

Supplementary tables

[Begins on the next page]

Date (UTC)	Event description	Source
2020-01-29	China Ministry of Science and Technology notice encouraging scientists to fight the epidemic and publish in Chinese journals	https://m.sohu.com/a/369721616_120059213/
2020-02-25	China CDC notice on Covid-19 publications and data sharing	https://www.documentcloud.org/documents/7340336-China-CDC-Sup-Regs.html
2020-03-02	Wuhan University press release on the nanopore paper	https://web.archive.org/web/20211203030758/https://news.whu.edu.cn/info/1002/57753.htm
2020-03-03	Notice by the Chinese Minister of Science and Technology on Covid-19 scientific research	https://www.documentcloud.org/documents/7340337-State-Research-regulations.html
2020-03-04	Wang *et al.* nanopore paper sent to medRxiv	https://www.medrxiv.org/content/10.1101/2020.03.04.20029538v1.article-info
2020-03-06	Wang *et al.* (2020a) nanopore paper posted on medRxiv	https://www.medrxiv.org/content/10.1101/2020.03.04.20029538v1.article-info
2020-03-16	PRJNA612766 submitted to SRA, SUB7147304	https://justthenews.com/sites/default/files/2022-03/nih-foia-request-56712_redacted.pdf
∼2020-03-31	Farkas *et al.* download of SRA metadata	https://peerj.com/articles/9255/
2020-04-03	Wang *et al.* nanopore paper received by *Small*	https://onlinelibrary.wiley.com/doi/epdf/10.1002/smll.202002169
2020-04-17	Application filed by Wuhan Zhenxi Medical Laboratory Co Ltd for patent related to the nanopore paper	https://patents.google.com/patent/CN111662958A/zh
2020-05-27	Wang *et al.* nanopore paper revision received by *Small*	https://onlinelibrary.wiley.com/doi/epdf/10.1002/smll.202002169
2020-06-01	Wang *et al.* nanopore paper accepted by *Small*	Feb 2024 email from Wiley's Integrity Assurance & Case Resolution team to FD
2020-06-09	Proofs of the Wang *et al.* nanopore paper sent to the authors	Feb 2024 email from Wiley's Integrity Assurance & Case Resolution team to FD; and https://www.youtube.com/watch?v=UA2P8hlurlQ&t=4606s
2020-06-16	Authors request withdrawal of SUB7147304 (PRJNA612766)	https://justthenews.com/sites/default/files/2022-03/nih-foia-request-56712_redacted.pdf
2020-06-17	PRJNA612766 withdrawn	https://justthenews.com/sites/default/files/2022-03/nih-foia-request-56712_redacted.pdf
2020-06-24	Wang *et al.* (2020b) nanopore paper published online at *Small*	https://onlinelibrary.wiley.com/doi/epdf/10.1002/smll.202002169
2020-06-28	Wuhan University tweets about the publication of Wang *et al.* (2020b) in *Small*	https://x.com/WHU_1893/status/1277218113642086402
2020-09-15	Publication of patent CN111662958A	https://patents.google.com/patent/CN111662958A/zh

Table S1: Timeline of 2020 events related to Wang *et al.*'s study and sequencing data. The dates are written in the YYYY-MM-DD format.

Position	3171	8782	18060	28144*	29095*
Lineage B (Wuhan-Hu-1)	T	C	C	T	C
Lineage A	T	**T**	C	**C**	C
Bloom 1: A+C18060T	T	**T**	**T**	**C**	C
Bloom 2: A+C29095T	T	**T**	C	**C**	**T**
Bloom 3: A+T3171C	**C**	**T**	C	**C**	C

Table S2: Substitutions in the different lineages and Bloom's proposed roots. We use Wuhan-Hu-1 as reference. The positions highlighted with a star (*) are covered in the Wang *et al.* (2020b) sequences. The lineage defined by A+C18060T is referred to as "proCoV2" by Bloom (2021), following previous analysis (Kumar *et al.*, 2021). Since the original "proCoV2" had three additional substitutions in the Kumar *et al.* preprint, we avoid this nomenclature.

ID	Age	Sex	Link	Onset	Hospitalization
SZ01	65	F	Mother of SH03	2020-01-03	2020-01-10
SZ02	66	M	Father of SH03	2020-01-04	2020-01-10
SZ03	37	F	Daughter of SH01 and SH02	2020-01-09	2020-01-11
SZ04	36	M	Son in law of SH01 and SH02	2020-01-05	2020-01-11
SZ05	10	M	Grandson of SH01 and SH02		2020-01-11
SZ06	63	F	Mother of SH04		2020-01-11

Table S3: Patients in the early January 2020 Shenzhen cluster. Hospitalization refers to the date of admission at HKU-SZH. Metadata from Chan *et al.* (2020).

Accession GISAID	ID	Collection	Source	Bloom label
EPI_ISL_406592	SZ01	2020-01-13	Yang *et al.*	
EPI_ISL_403933	SZ01	2020-01-15	Kang *et al.* (2020)	Guangdong patients
EPI_ISL_406030	SZ02	2020-01-10	Chan *et al.* (2020)	Guangdong patients
EPI_ISL_406593	SZ02	2020-01-13	Yang *et al.*	other China
EPI_ISL_403932	SZ02	2020-01-14	Kang *et al.* (2020)	Guangdong patients
EPI_ISL_405839	SZ05	2020-01-11	Chan *et al.* (2020)	other China
EPI_ISL_403935	SZ06	2020-01-15	Kang *et al.* (2020)	Guangdong patients

Table S4: Sequences in the early January 2020 Shenzhen cluster. Yang *et al.* are Yang Yang, Chenguang Shen, Li Xing, Zhixiang Xu, Haixia Zheng, Yingxia Liu, as listed on GISAID; we have not found a specific article presenting the sequences. The ID column corresponds to patient IDs introduced in Table S3.

Supplementary tables

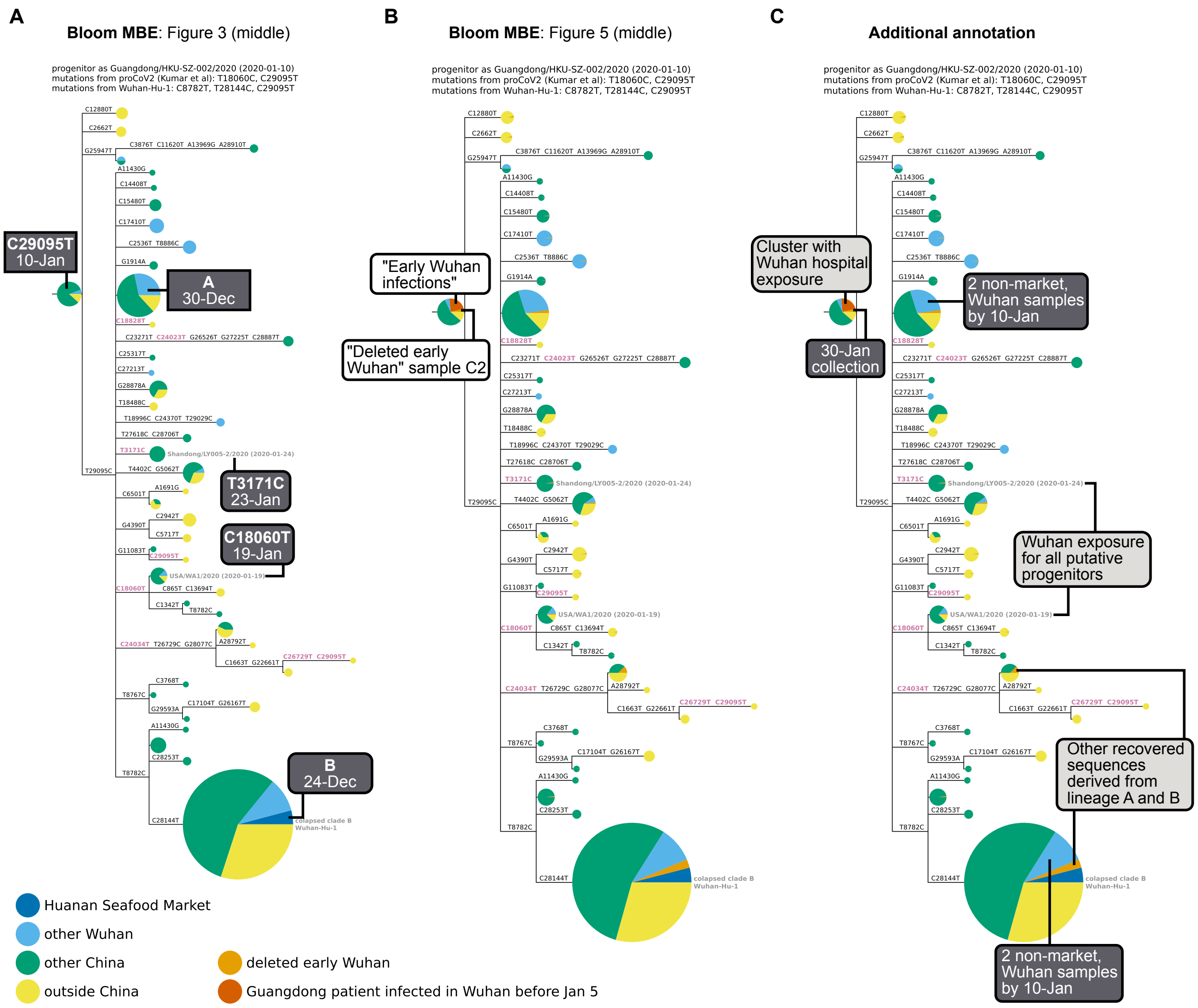

Figure S1: The proposed progenitor A+C29095T is not supported by a more completely annotated phylogenetic tree of sequences from samples collected before February 2020. This figure is adapted from the middle panels of Figure 3 and Figure 5 from Bloom's article, with our added annotations in rounded rectangles. Additional annotations include sample collection date metadata (dark gray), other metadata (light gray), and phrases used by Bloom to describe subsets of sequences (white). (A) The conundrum Bloom sought to resolve is illustrated by annotating lineage A, lineage B, and three proposed progenitor genotypes by the date of the earliest sample collection date in each node; samples more similar to related bat coronaviruses (Lineage A with one of T3171C, C18060T, or C29095T) were sampled later than samples from lineage A and lineage B. (B) To support A+C29095T as a progenitor genotype, Bloom added recovered sequences to the phylogenetic tree in positions consistent with their composition. He annotated two fractions of the A+C29095T progenitor node as being linked to Wuhan and sampled early relative to samples collected before February 2020: a fraction corresponding to one recovered sequence (orange) and a fraction corresponding to patients in Guangdong province with Wuhan travel history (red). (C) Additional annotation challenges the conclusion that A+C29095T is a likely progenitor genotype. Recovered sequences were from samples collected on 30 January (A+C29095T progenitor node, and in other nodes in the tree, two of which are annotated as including sequences in lineages derived from lineage A and lineage B). Early sequences of A+C29095T collected in Guangdong province correspond to a single cluster with exposure to a Wuhan hospital in late December 2019 i.e. this is a well studied cluster and not independent pieces of supporting data. Wuhan exposure history is extremely common in pre-February sequences including for lineage A, lineage B, and the three proposed progenitor genotypes. In contrast, sequences in both lineage A and lineage B with no link to Huanan market were collected prior to the earliest collection date for A+C29095T.

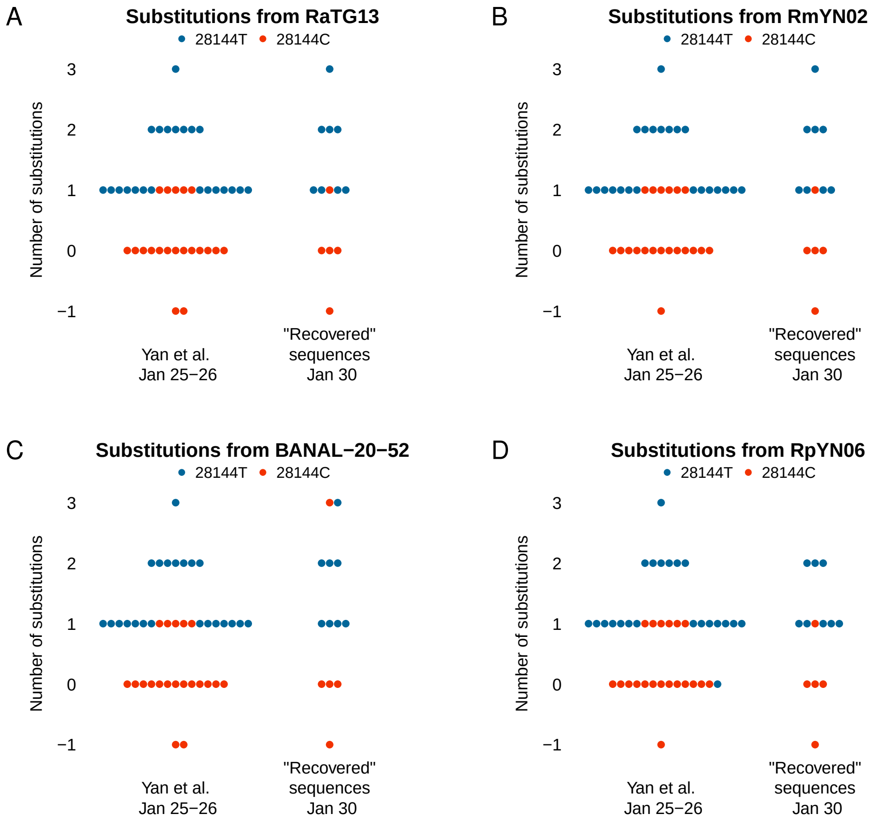

Figure S2: Equivalent of Figure 2, changing the outgroup comparator, shown as title of each panel.

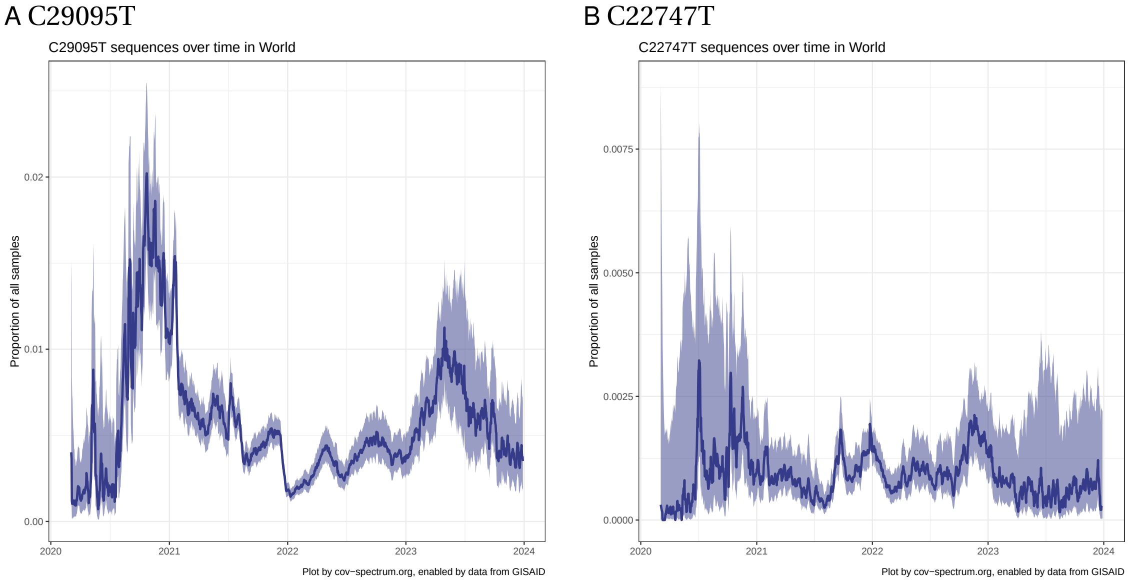

Figure S3: Proportion of sequences with C29095T and with C22747T among all sequences available on GISAID from 1 March 2020 through the end of 2023. Plots generated by CoV-Spectrum (Chen *et al.*, 2022), from `https://cov-spectrum.org/explore/World/AllSamples/from%3D2020-03-01%26to%3D2024-01-01/variants?nucMutations=C29095T&` and `https://cov-spectrum.org/explore/World/AllSamples/from%3D2020-03-01%26to%3D2024-01-01/variants?nucMutations=C22747T&` . Note the different vertical axis scales.

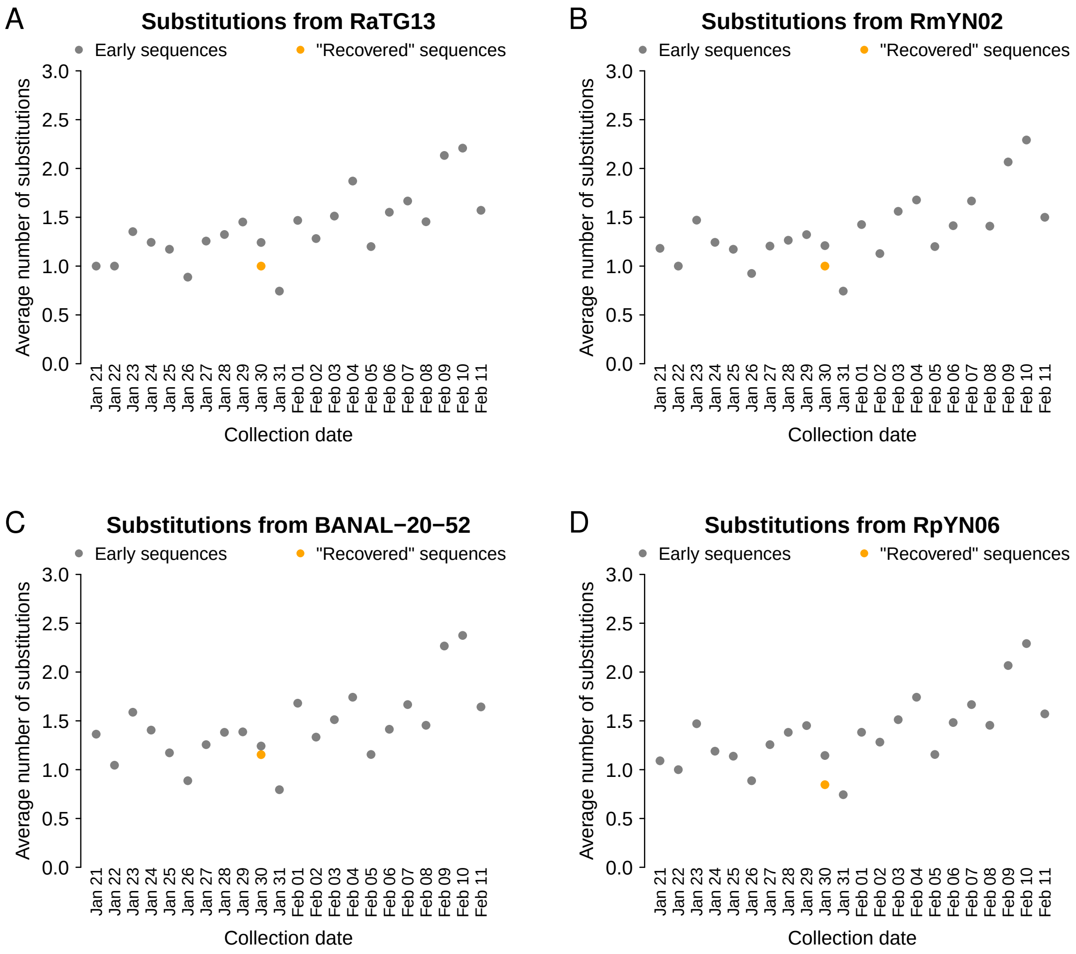

Figure S4: Average number of substitutions from various bat SARS-like outgroup comparators (relative to lineage A, or, equivalently, lineage A+C18060T), over SARS-CoV-2 nucleotides 21,570–29,550, comparing all available sequences (gray) to Wang *et al.* (2020b)'s sequences (orange).

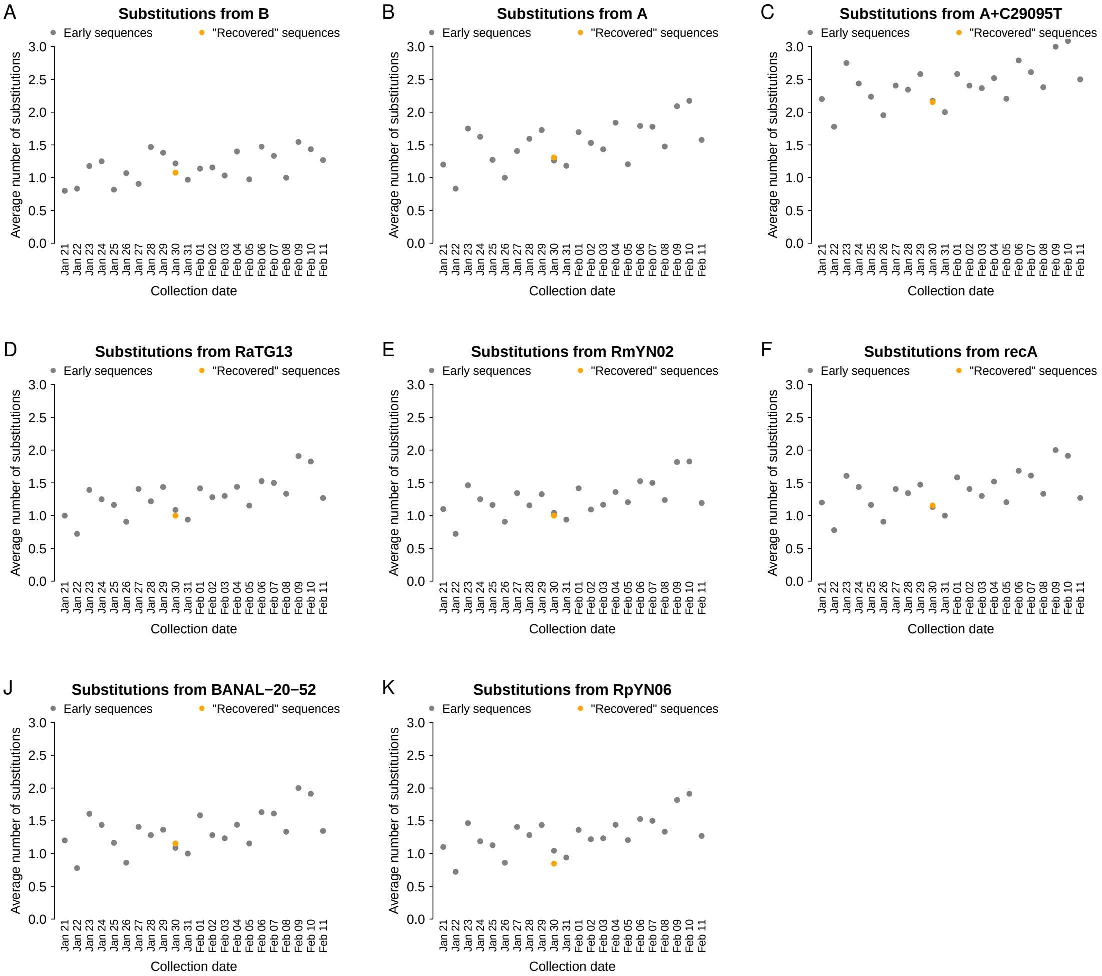

Figure S5: Equivalent of Figure S4, using a more curated dataset (Pekar *et al.*, 2022), to which we added more recently published sequences (Lv *et al.*, 2024).

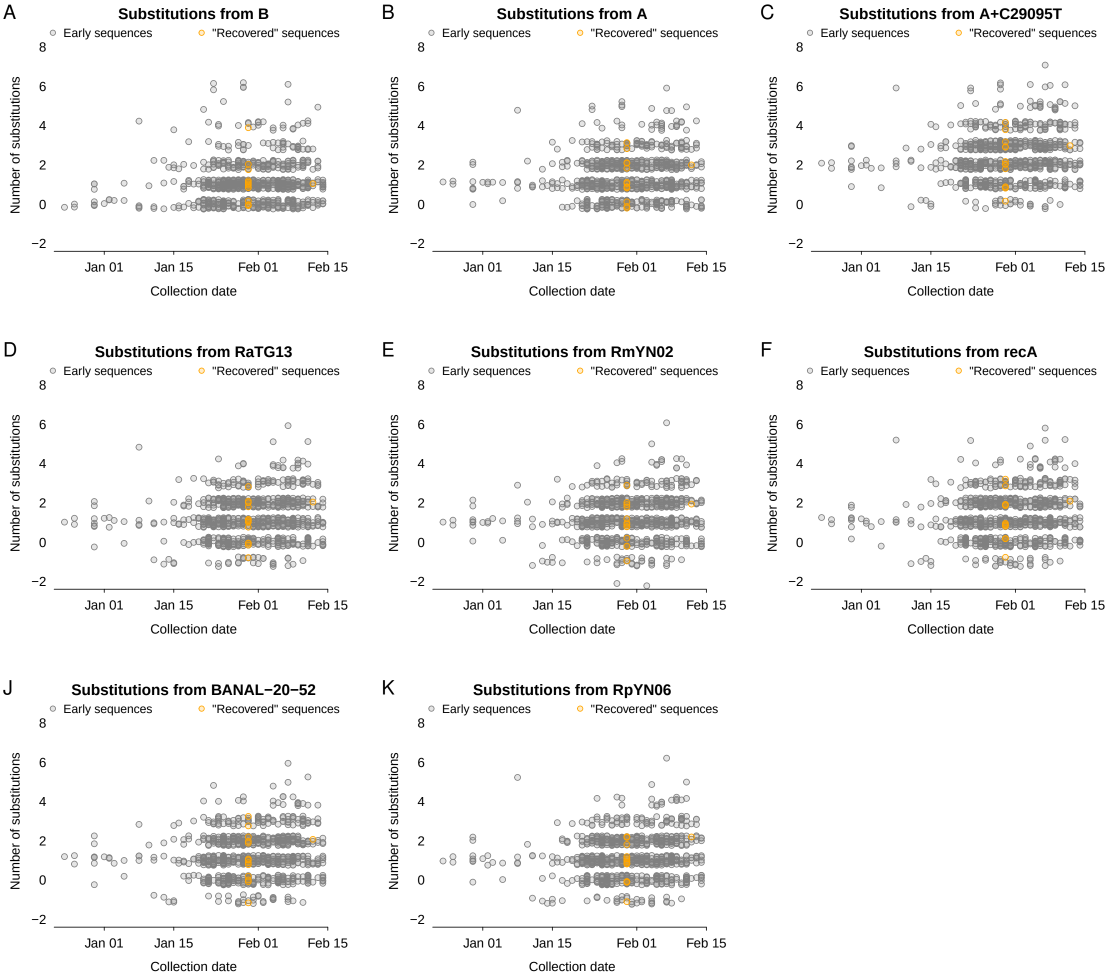

Figure S6: Equivalent of Figure S5, showing all points instead of averages and over a wider time window. The outgroup comparators are shown as panel titles.

References

Bloom JD. 2021. Recovery of Deleted Deep Sequencing Data Sheds More Light on the Early Wuhan SARS-CoV-2 Epidemic. Molecular Biology and Evolution. 38:5211–5224. doi:10.1093/molbev/msab246.

Chan JFW, Yuan S, Kok KH, To KKW, Chu H, Yang J, Xing F, Liu J, Yip CCY, Poon RWS *et al.* 2020. A familial cluster of pneumonia associated with the 2019 novel coronavirus indicating person-to-person transmission: A study of a family cluster. The Lancet. 395:514–523. doi:10.1016/S0140-6736(20)30154-9.

Chen C, Nadeau S, Yared M, Voinov P, Xie N, Roemer C, Stadler T. 2022. CoV-Spectrum: Analysis of globally shared SARS-CoV-2 data to identify and characterize new variants. Bioinformatics. 38:1735–1737. doi:10.1093/bioinformatics/btab856.

Farkas C, Fuentes-Villalobos F, Garrido JL, Haigh J, Barría MI. 2020. Insights on early mutational events in SARS-CoV-2 virus reveal founder effects across geographical regions. PeerJ. 8:e9255. doi:10.7717/peerj.9255.

Kang M, Wu J, Ma W, He J, Lu J, Liu T, Li B, Mei S, Ruan F, Lin L *et al.* 2020. Evidence and characteristics of human-to-human transmission of SARS-CoV-2. Preprint. Epidemiology. doi:10.1101/2020.02.03.20019141.

Kumar S, Tao Q, Weaver S, Sanderford M, Caraballo-Ortiz MA, Sharma S, Pond SLK, Miura S. 2021. An Evolutionary Portrait of the Progenitor SARS-CoV-2 and Its Dominant Offshoots in COVID-19 Pandemic. Molecular Biology and Evolution. 38:3046–3059. doi:10.1093/molbev/msab118.

Lv JX, Liu X, Pei YY, Song ZG, Chen X, Hu SJ, She JL, Liu Y, Chen YM, Zhang YZ. 2024. Evolutionary trajectory of diverse SARS-CoV-2 variants at the beginning of COVID-19 outbreak. Virus Evolution. p. veae020. `https://academic.oup.com/ve/article/10/1/veae020/7619252`.

Pekar JE, Magee A, Parker E, Moshiri N, Izhikevich K, Havens JL, Gangavarapu K, Malpica Serrano LM, Crits-Christoph A, Matteson NL *et al.* 2022. The molecular epidemiology of multiple zoonotic origins of SARS-CoV-2. Science. 377:960–966. doi:10.1126/science.abp8337.

Wang M, Fu A, Hu B, Tong Y, Liu R, Gu J, Liu J, Jiang W, Shen G, Zhao W *et al.* 2020a. Nanopore target sequencing for accurate and comprehensive detection of SARS-CoV-2 and other respiratory viruses. Preprint. Infectious Diseases (except HIV/AIDS). doi:10.1101/2020.03.04.20029538.

Wang M, Fu A, Hu B, Tong Y, Liu R, Liu Z, Gu J, Xiang B, Liu J, Jiang W *et al.* 2020b. Nanopore Targeted Sequencing for the Accurate and Comprehensive Detection of SARS-CoV-2 and Other Respiratory Viruses. Small. 16:2002169. doi:10.1002/smll.202002169.
